# Volvulus of Small Bowel in a Case of Simple Meconium Ileus

**Published:** 2011-03-10

**Authors:** Kanchan Kayastha, Bilal Mirza, Afzal Sheikh

**Affiliations:** Department of Pediatric Surgery, The Children's Hospital and the Institute of Child Health Lahore, Pakistan

**Keywords:** Meconium ileus, Volvulus, Complications

## Abstract

Meconium ileus is one of important causes of neonatal intestinal obstruction. Many patients respond well to nonsurgical management with enemas, however, few patients may develop complications in the postnatal period thus requiring urgent operation. A 2 day old newborn presented with clinical features of intestinal obstruction. There was a suspicion of meconium ileus. Contrast x-ray with gastrografin enema was suggestive of unused colon with beaded appearance. Patient had to be surgery as repeated enemas did not improve the condition and progressive abdominal distension occurred. At exploration twist of the dilated, meconium filled loop of small bowel found. De-twisting of the volvulus done and Bishop Koop ileostomy fashioned. Patient made an uneventful recovery. Stoma was closed six months later.

## INTRODUCTION

Meconium ileus can be simple or complicated. Simple meconium ileus may progress to complicated meconium ileus by volvulus and/or perforation of the meconium filled loop. These events usually occur in-utero, however, they have also been observed in postnatal period but with extreme rarity [1, 2].

The recommended treatment of simple meconium ileus is evacuation of the thick inspissated meconium with enemas. In cases where postnatal complication occurs, urgent surgical interventions are required [1]. We are presenting a case of simple meconium ileus that developed volvulus of the small bowel, laden with thick inspissated meconium.

## CASE REPORT

A two days old male baby weighing 2.5 kg presented with abdominal distension, failure to pass meconium and biliary emesis since birth. The baby was born by spontaneous vaginal delivery at home. According to the mother the patient was born with a distended abdomen and passed white pellets per rectally. The abdominal distension gradually worsened with bilious vomiting after every attempt at feeding. General physical examination revealed a lethargic and ill looking baby with obvious respiratory distress and abdominal distension. He was febrile with temperature of 100 °F; respiratory rate 45/min, and pulse 150/min. Bowel loops were visible. A per-rectal examination revealed meconium beads. A preoperative diagnosis of neonatal intestinal obstruction secondary to meconium ileus, with a differential of distal intestinal atresia, was made.

The newborn was resuscitated with intravenous infusion. A nasogastric tube was passed and gastric aspiration done. The neonate was given injection Vit. K and intravenous antibiotics started. X-ray abdomen showed dilated bowel loops. Contrast x-rays with gastrografin enema delineated a small caliber colon with filling defects as of meconium beads (Fig. 1). As condition of the baby did not improve an exploratory laparotomy was performed. At laparotomy volvulus of distended small bowel found. The colour of the involved segment was dark and appeared congested (Fig. 2). The volvulus was corrected by untwisting the mesentery. The involved gut was hugely distended due to the presence of thick tenacious meconium. The thick meconium was concentrated in distal small bowel whereas the colon was packed with small beads. An enterotomy was made at the most distended portion (distal ileum) and irrigated with diluted gastrografin. Gentle milking was then performed to remove the meconium. The large gut also washed in the similar way. Bishop Koop chimney was made after resecting a small portion of small bowel which was of doubtful viability.

**Figure:**
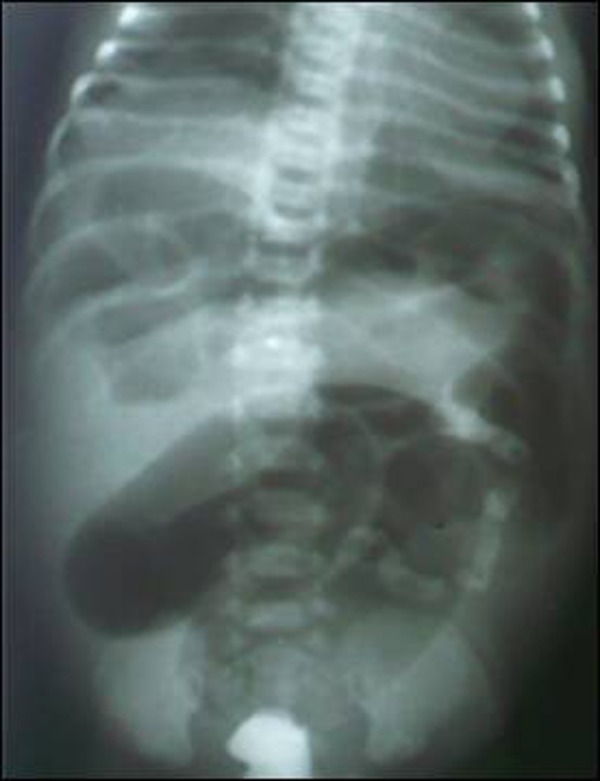
Figure 1: X-ray abdomen erect with gastrografin enema, showing small caliber of colon and filling defects

**Figure:**
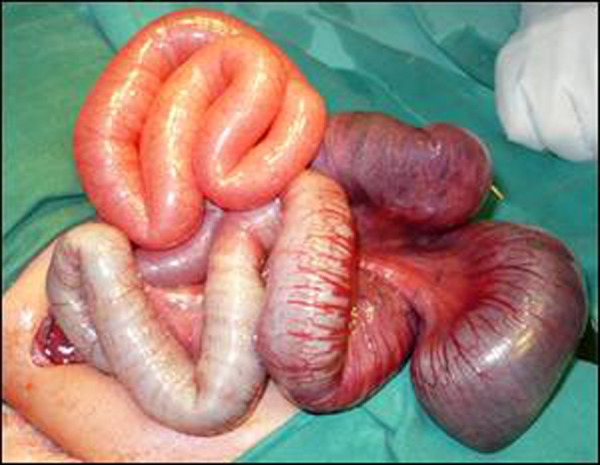
Figure 2: Operative view of twisted small bowel. The discolored and hugely distended bowel is obvious

The postoperative recovery was uneventful. NG tube was removed on 4th post-op day with oral feed on following day and discharged on 7th postoperative day. The patient has an uneventful follow up after Bishop-Koop stoma closure (at the age of 6 months).

## DISCUSSION

Meconium ileus is characterized by presence of thick and tenacious meconium in small intestine causing neonatal intestinal obstruction. It accounts for 33% of neonatal small bowel obstruction. There is intra-luminal accumulation of highly viscid and tenacious meconium which begins in utero. The cause of increased viscosity of meconium is referred to abnormally high amount of albumin and other mucoproteins. There is strong association with cystic fibrosis in west but not described in our part of world [1].

Meconium ileus can be uncomplicated (simple meconium ileus) or complicated. Uncomplicated meconium ileus presents at birth with abdominal distension and failure to pass meconium. Less than half of them present with complications. The complications in meconium ileus may occur either prenatally or postnatally. In-utero complications include volvulus with perforation, meconium peritonitis, giant meconium cyst or atresia etc. Due to massive bowel distension volvulus, perforation and peritonitis can also occur in the postnatal period [2, 3]. In our case patient initially suspected to have simple meconium ileus which then got complicated. The ex-utero volvulus is a rare event.

A non-operative management with gastrografin enema is often successful. This technique is useful in evacuating the thick and sticky meconium from the gut under fluoroscopy. In complicated meconium ileus and in cases where simple meconium ileus does not resolve or complicates an immediate surgery is advised [1, 4]. We initially tried gastrografin enema but due to progressive abdominal distension and rapid deterioration of the clinical condition an emergency laparotomy was performed.

The presentation of simple meconium ileus with volvulus can be missed if not taken into consideration. Early surgical intervention is required to manage such a situation.

## Footnotes

**Source of Support:** Nil

**Conflict of Interest:** None declared
